# A GFP-fusion coupling FACS platform for advancing the metabolic engineering of filamentous fungi

**DOI:** 10.1186/s13068-018-1223-8

**Published:** 2018-08-24

**Authors:** Guokun Wang, Wendi Jia, Na Chen, Ke Zhang, Lixian Wang, Pin Lv, Ronglin He, Min Wang, Dongyuan Zhang

**Affiliations:** 1 Tianjin Institute of Industrial Biotechnology, Chinese Academy of Sciences, Tianjin, 300308 People’s Republic of China; 2 Tangshan Academy of Agricultural Sciences, Tangshan, 063001 People’s Republic of China

**Keywords:** Fatty alcohol, Filamentous fungi, Fluorescence-activated cell sorting, Metabolic engineering, *Trichoderma reesei*

## Abstract

**Background:**

The filamentous fungus *Trichoderma reesei*, the most widely used cellulase producer, also has promising applications in lignocellulose-based biorefinery: consolidated bioprocessing for the production of high value-added products. However, such applications are thwarted by the time-consuming metabolic engineering processes (design–build–test–learn cycle) for *T. reesei*, resulted from (i) the spore separation-mediated purification as the multinucleate hyphae, (ii) transformant screening for high expression levels since unavailable of episomal expression system, and (iii) cases of inexpressible heterologous proteins.

**Results:**

In this study, a GFP-fusion coupled fluorescence-activated cell sorting (FACS) platform was established to speed up the build and test process of the DBTL cycle, by enabling rapid selection for expressible heterologous genes and bypassing both laborious spore separation and transformant screening. Here, the feasibility of flow cytometry in analyzing and sorting *T. reesei* cells harboring GFP-fused expressible protein was proven, as well as the application of the platform for constitutive promoter strength evaluation. As a proof-of-concept, the platform was employed to construct the first *T. reesei* strain producing fatty alcohol, resulting in up to 2 mg hexadecanol being produced per gram biomass. Pathway construction was enabled through rapid selection of functional fatty acyl-CoA reductase encoding gene *Tafar1* from three candidate genes and strains with high expression level from spore pools. As a result of using this method, the total costed time for the build and test cycle using *T. reesei,* subsequently, reduced by approx. 75% from 2 months to 2 weeks.

**Conclusion:**

This study established the GFP-fusion coupling FACS platform and the first filamentous fungal fatty alcohol-producing cell factory, and demonstrated versatile applications of the platform in the metabolic engineering of filamentous fungi, which can be harnessed to potentially advance the application of filamentous fungi in lignocellulose-based biorefinery.

**Electronic supplementary material:**

The online version of this article (10.1186/s13068-018-1223-8) contains supplementary material, which is available to authorized users.

## Background

Cell factories are promising alternatives for the production of bulk chemicals [1, 2], transport fuel [3, 4], and natural products [5, 6] currently produced based on petroleum industry or plant-derived extraction. Construction of efficient cell factories is mainly achieved through cycles of the design–build–test–learn (DBTL) process, which typically involves reconstituting heterologous metabolic pathways and rewiring native cellular metabolism [7]. Due to the advantage of a well-understood cellular metabolism and a comprehensive genetic manipulation platform, both *Escherichia coli* [8–10] and *Saccharomyces cerevisiae* [11–13] predominate as hosts for rapid cell factory construction. However, their application in industry may be limited by expensive feedstock consumption (glucose, glycerol, etc.), especially when economic pressures on TRY (titer, rate, and yield) are high, such as in the case of low-priced bulk chemical and fuel production. Cost-effective bioprocessing, subsequently, necessitates the generation of cell factory utilizing inexpensive, abundant, and renewable feedstocks such as CO_2_ [14] and lignocellulose [15]. To develop the direct lignocellulose utilization-based biorefineries, many efforts have been made on strain improvement, mainly using yeast, for cellulose degradation (consolidated bioprocessing [16, 17]) and xylose utilization [18, 19]. In contrast, the potential use of native cellulose-degrading microorganisms such as *Clostridium* spp. [20, 21] and filamentous fungi, for high-value chemical production, has been scarcely explored. The Sordariomycete fungus *Trichoderma reesei* is the most widely used cellulase producer in both academic investigations and industrial applications [22]. As it demonstrates a great capability for degrading cellulose, *T. reesei,* subsequently, has huge potential in consolidated bioprocessing, to convert recalcitrant and abundant cellulose into value-added products via single microorganism-based fermentation [23].

Rational engineering is essential for strain improvement, especially for manufacturing non-native products, which involves introducing and optimizing heterologous pathways. Through this approach of systematic and rational metabolic engineering, much progress has been made in *T. reesei* for improving its cellulase-producing capacity, by optimizing its native secretory pathway [24] or by introducing heterologous glucosidase for efficient enzymatic cellulose degradation [25]. Nevertheless, there still exist many drawbacks hindering rational strain improvement in filamentous fungi. This includes the current need to invest significantly more time into genetic engineering efforts (2 months versus less than 1 week for model organisms), which nonetheless, results in low efficiency heterologous protein expression (Additional file 1: Fig. S1). This genetic engineering approach, which is complexified by the unavailability of episomal plasmids, also involves spore separation of positive transformants and subsequent strain evaluation without high-throughput screening, leading to the overall strain build and test process of *T. reesei* being time-consuming, effort-intensive, and generally inefficient (Additional file 1: Fig. S1). A means for simplifying this time-consuming genetic engineering process, as well as readily excluding effort-intensive unsuccessful heterologous protein expression in filamentous fungi, thus need to be addressed.

**Fig. 1:**
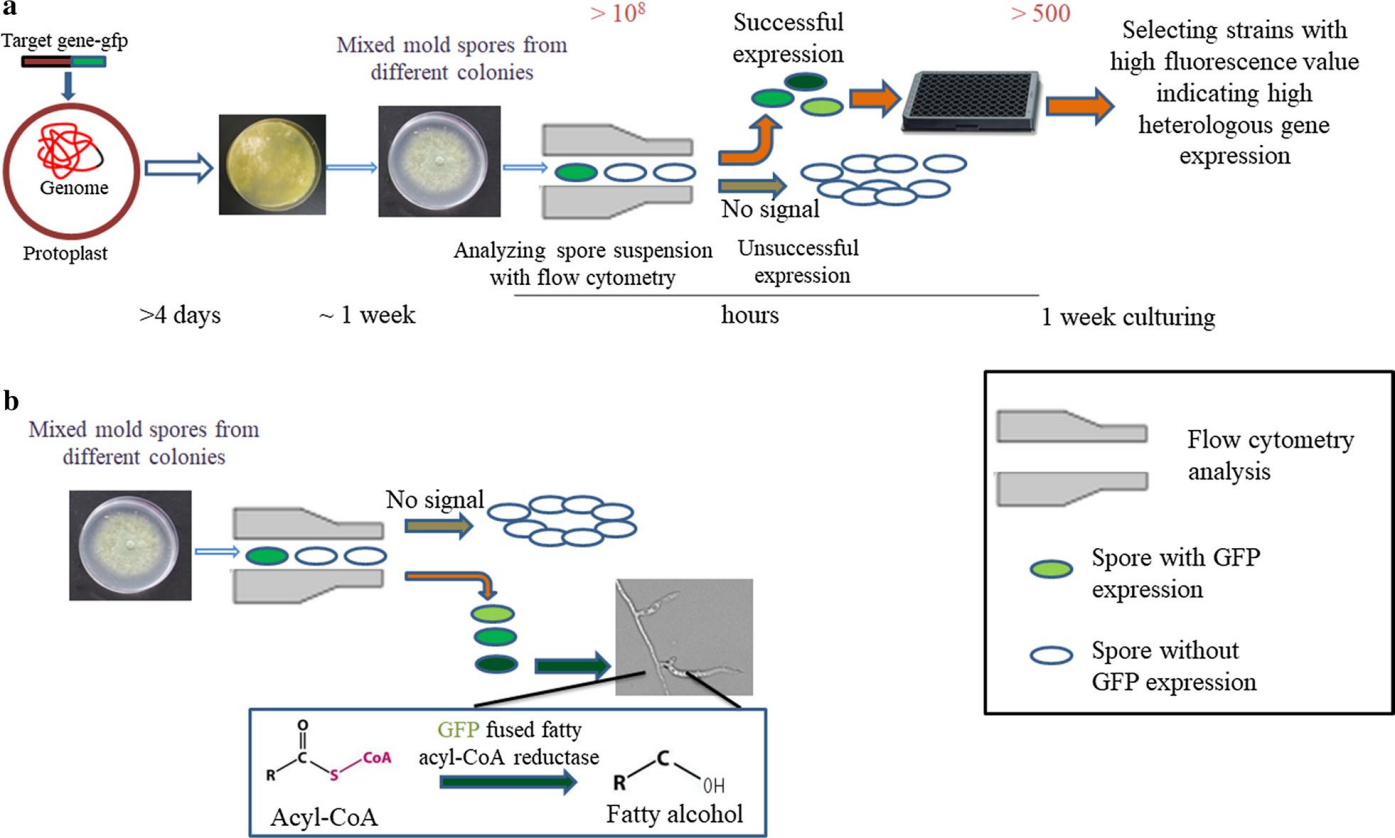
Schematic illustrating the GFP-fusion coupling FACS platform accelerating the heterologous gene expression process in *T. reesei.* **a** *gfp* gene was fused to the target gene and acted as the indicator for successful expression of heterologous genes and for spore separation through an FACS platform. Heterologous genes of unsuccessful expressions would be excluded following flow cytometry analysis, since no fluorescence was detected. For genes with successful expression, strains with gene expressed at expected levels can be rapidly obtained from a large candidate pool through cell sorting, as well as an additional confirmation of hyphae fluorescence on 96-well plate cultures. Time and numbers marked in the figure indicated the time and numbers of selected transformants in each procedure. **b** Cellular fatty acyl-CoA could be converted to fatty alcohol by functional fatty acyl-CoA reductase (FAR). The FAR viability was monitored using FAR fused with GFP with FAR expression levels being reflected by GFP fluorescence intensity

Flow cytometry-based platforms have wide application in the analysis and sorting of single cells mainly due to their ability to screen and, if necessary, sort a large number of cells quickly. We, therefore, leveraged this type of high-throughput platform, using specifically GFP-fusion coupled fluorescence-activated cell sorting (FACS), to accelerate the genetic engineering of *T. reesei.* Using this method, we were thus able to rapidly select expressible heterologous gene and purify transformants with high expression levels (Fig. 1). As a proof-of-concept for using this platform with filamentous fungi, we showed that it was possible to rapidly screen *T. reesei* strains with high expression levels of viable fatty acyl-CoA reductase (Fig. 1). In doing so, we constructed the first viable filamentous fungi cell factory for the production of fatty acid derivatives.

## Results

### Flow cytometry as a robust tool for analyzing filamentous fungi in high throughput

**Fig. 2:**
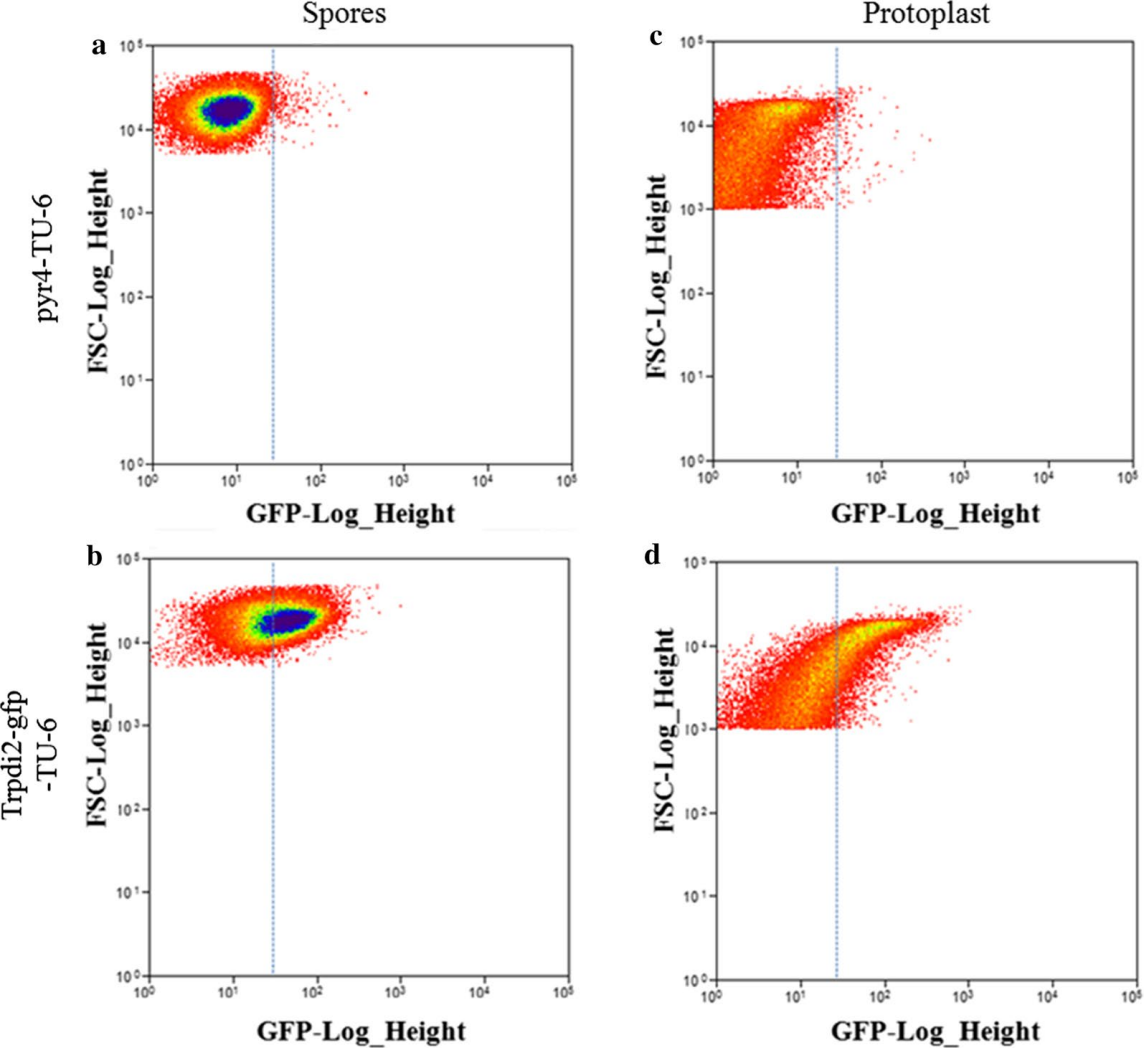
Flow cytometry analysis of *T. reesei* cells harboring *gfp*-fused gene construct. *Trpdi2* was selected as the homologous gene, which was confirmed as expressing functionally, for feasibility of flow cytometry analysis of *T. reesei* cells with gene–*gfp*-fusion construct. Both spores (**a**, **b**) and protoplast (**c**, **d**, generated from hyphae by enzymatic degradation) were utilized for the evaluations. *pyr4*-TU-6 strain served as the negative control in analyzing the fluorescence distribution of *Trpdi2*-*gfp*-TU-6 strain’s cell population. 100,000 or 30,000 cells were analyzed for spore or protoplast samples, respectively. Dashed and light-blue lines marking the same value of GFP-Log_Height were for direct comparisons of results from different panels. *FSC* forward scatter

To test the feasibility of flow cytometry in analyzing filamentous fungi, we tested *T. reesei* based on both its cell shape and fluorescence, as well as testing the gene expressibility, we applied flow cytometry to analyze spores and protoplasts of *pyr4*-TU-6 strain and *Trpdi2*-*gfp*-TU-6 strain expressing GFP-fused homologous TrPDI2 (protein disulfide isomerase, [24]). The latter strain was selected as demonstration not only because of the capability of emitting fluorescence but also for the purpose of testing the performance of strain carrying target gene–gfp construct (subsequently used for rapid heterologous gene evaluation), since TrPDI2 is a expressible native gene. In contrast to the control strain *pyr4*-TU-6, wherein no GFP was present, the cell population of *Trpdi2*-*gfp*-TU-6 showed much higher fluorescence levels (Fig. 2). This suggested that flow cytometry was able to identify *T. reesei* cells (both spores and protoplasts) and discern according to their difference in fluorescence intensity, consistent with the previous studies proving the application feasibility of flow cytometry on *T. reesei* germinating spores [26, 27]. And more importantly, our results confirmed that GFP-fusion construct was suitable for the rapid confirmation of gene expressibility.

### Promoter strength evaluation by flow cytometry analysis

**Fig. 3:**
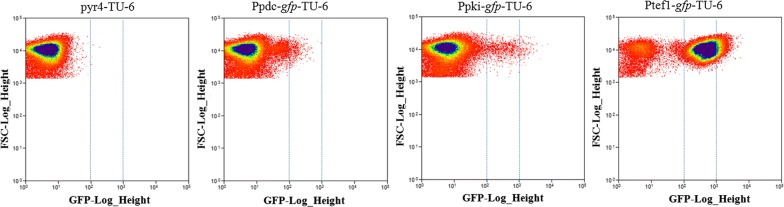
Flow cytometry analysis of *T. reesei* spores constitutively expressing *gfp.* GFP was driven by three strong promoters on originally constructed plasmids designed for convenient cloning (multiple cloning site) and multiple segment assembly (BioBrick assembly). These plasmids were co-transformed into TU-6 with P19-*pyr4* for transformant’s spore collection. 100,000 cells were analyzed for each sample. Two batches of analysis were performed, with the same indications of the variation between samples. Dashed and light-blue lines marking the same value of GFP-Log_Height were for direct comparisons of results from different panels. *FSC* forward scatter

After confirming the proof of principle of our flow cytometry platform for measuring gene expressibility, we then tested the feasibility of this analysis tool for evaluating constitutive promoter strength, the basal and crucial element for stable gene expression. Strength assessment of promoters, both heterologous and homologous, was mainly performed with RT-PCR, a reliable but relatively low-throughout method. We, subsequently, analyzed the GFP-promoting strength of three previously reported promoters: glucose concentration responsive *pdc* promoter [28], constitutive pyruvate kinase (*pki*) promoter [29], and constitutive *tef1* promoter with flow cytometry. Here, spores of each transformant pool showed two cell populations: fluorescence-positive and fluorescence-negative (Fig. 3), indicating the existence of false-positive transformants by co-transformation. The overall spores’ fluorescence distribution showed that the fluorescence of spores expressing *gfp* under *tef1* and *pki* promoters (with similar fluorescence range) was higher compared to that under *pdc* promoter (Fig. 3), while the quantitative analysis on median fluorescence value indicated the slightly lower strength of *pki* promoter compared to *tef1* promoter (Additional file 1: Fig. S2). These results on constitutive *tef1* and *pki* promoters, were consistent with previously published transcriptome data showing relatively comparable expression levels of these three genes [30] (also briefed in Additional file 1: Fig. S2). The relatively low strength of the *pdc* promoter (Fig. 3) detected via our flow cytometry method should be as this promoter’s high glucose concentration responsive property and that spores used for test were only formed under condition of low glucose concentration, that is when accessible nutrient in the medium was depleted. Our results, therefore, suggest that flow cytometry analysis is suitable for constitutive promoter strength evaluation.

### Applying flow cytometry for the rapid screening of viable heterologous genes and target strains

To obtain cells with high gene expression levels in high throughput, flow cytometry-based cell sorting is essential. We, therefore, evaluated the efficiency of cell sorting, according to both cell shape and fluorescence, for acquiring optimal strains of *T. reesei*. The viability of 20–50% of the sorted spores was observed under the condition used in this study. In contrast to this, protoplasts in our hands could be recovered at a much lower rate (less than 5%), mainly as the protoplast vulnerability for lacking the protection from cell wall. Therefore, spores were employed for further investigation for their characteristics of the direct and easy-to-operate fluorescence-emitting host (compared to protoplasts).

**Fig. 4:**
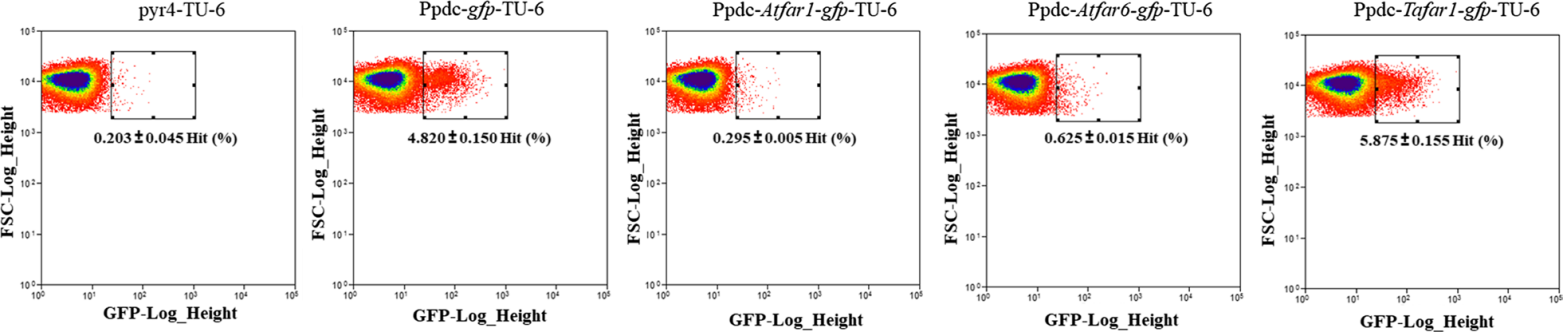
Flow cytometry analysis of functional viability of fatty acyl-CoA reductases (FARs) in *T. reesei.* Spores harboring heterologous genes encoding FARs driven by the *pdc* promoter were analyzed for the evaluation of functional viable FARs in *T. reesei*. *pyr4*-TU-6 and Ppdc-*gfp*-TU-6 strains were used as the negative and positive controls, respectively. Gates marking the same value range were defined according to the fluorescence distribution of positive cells of Ppdc-*gfp*-TU-6 spores. Numbers indicate the mean ratio of gate-defined spore number to that of the whole-cell population, and standard deviation was calculated from triplicate analysis on transformant spore pools. 100,000 cells were analyzed for each sample. Two batches of experiments were performed, and significant differences were only observed between *pyr4*-TU-6 and Ppdc-*Tafar1*-*gfp*-TU-6 for FAR-expressing spores. *FSC* forward scatter

We next applied flow cytometry-based analysis and sorting to *T. reesei*, to screen for functionally viable heterologous genes and strains that exhibited desired expression levels following pathway construction. We pursued the reconstruction of metabolic pathways for fatty alcohol production as an example, as these are a key type of aliphatic fatty acid derivatives, with wide applications in detergent, lubricant, and cosmetics. The biosynthesis of fatty alcohols derived from fatty acyl-CoA can be directly catalyzed by fatty acyl-CoA reductases (FARs), which has been utilized for the highest titer reported until now [31]. FAR thus is typically favored in metabolic engineering efforts to construct cell factories for fatty alcohol production. We, therefore, tested several previously reported fatty acyl-CoA reductases from plant *Arabidopsis thaliana* (*Atfar1* and *Atfar6* [32–34]) and barn owl *Tyto alba* (*Tafar1* [35]) for their functional viability in *T. reesei* (Fig. 1b). Fusion segments of *far*-*gfp* under the *pdc* promoter were first constructed and transformed into TU-6, generating a transformant spore pool for flow cytometry analysis. Compared to the negative control (*pyr4*-TU-6 spores), an additional spore population with high fluorescence intensity was observed in *gfp*-TU-6 (positive control) and the corresponding high fluorescence intensity region was designated as a gate for evaluating potential gene viability (Fig. 4). We found the results for spores containing plant FARs, *Atfar1*-*gfp*-TU-6 and *Atfar6*-*gfp*-TU-6, to resemble that of the negative control with no high fluorescence being present amongst the spore population. In contrast, however, our analysis of *Tafar1*-*gfp*-TU-6 spores demonstrated a significantly higher ratio in the defined region (gate) and a similar performance to the positive control (Fig. 4). This suggested that *Tafar1* can be expressed successfully and can potentially act as the catalytic intermediary converting fatty acyl-CoA to fatty alcohol in *T. reesei*.

**Fig. 5:**
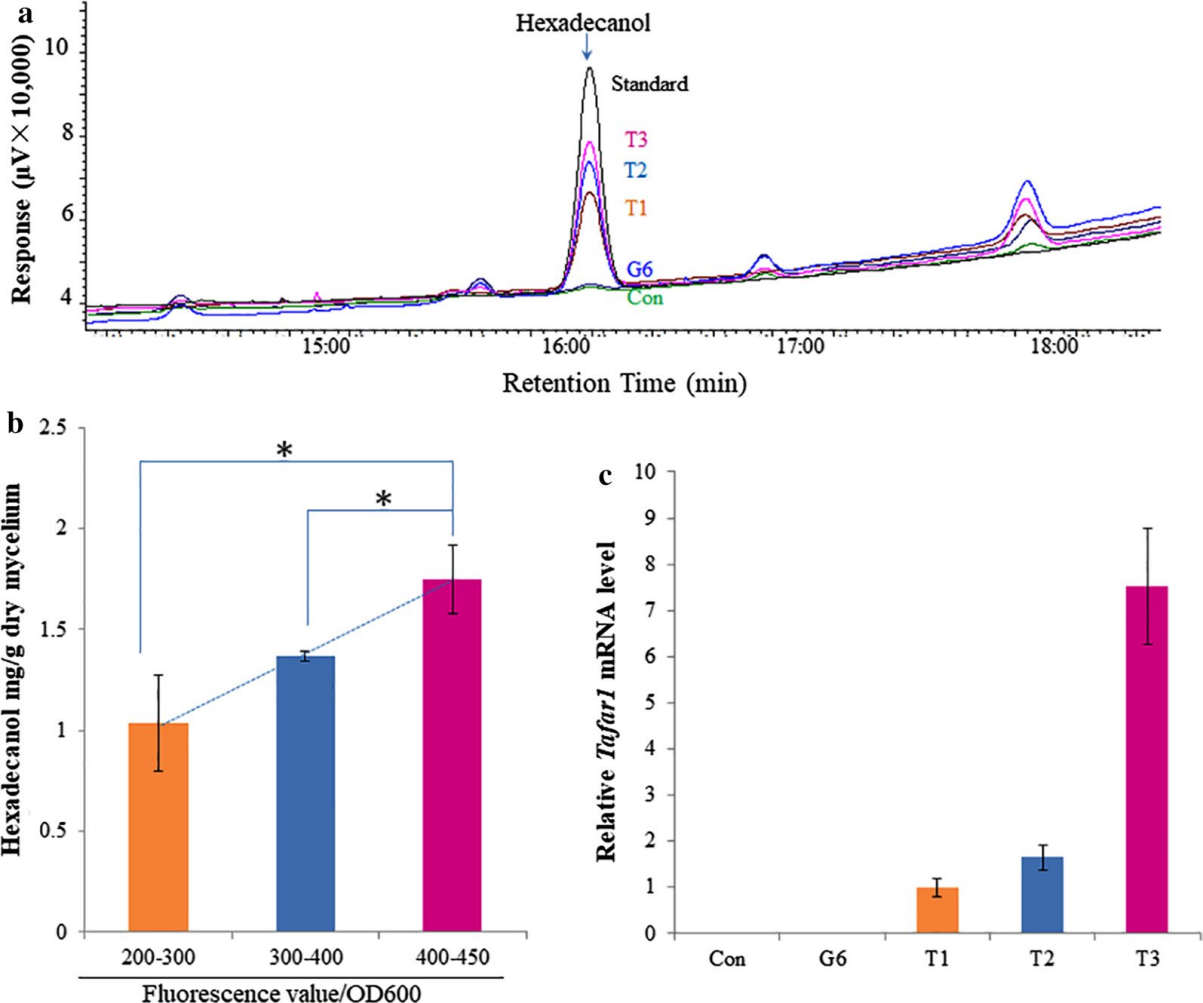
Correlation of TaFAR1-fused GFP intensity and fatty alcohol production in *T. reesei.* Flow cytometry-sorted spores were cultivated and subjected to fatty alcohol quantification (**a**) and hyphae fluorescence detection (**b**). Strains were grouped according to the fluorescence value/OD600 (**b**). 10 or 1 strain from each group was randomly selected for fatty alcohol quantification (**a**, **b**) or *Tafar1* expression detection (**c**), respectively. Results of hexadecanol yield are the mean of experiments on ten separate strains and error bars indicate standard deviations (*n* = 10 ± SD). Asterisks indicated a significant difference (*p* < 0.05) according to Student’s *t* test. Con: *pyr4*-TU-6 strain, G6: Ppdc-*gfp*-TU-6 strain, T1, T2, T3: Ppdc-*Tafar1*-*gfp*-TU-6 strains randomly selected from groups with different fluorescence value/OD600 (200–300, 300–400, 400–450), respectively

**Fig. 6:**
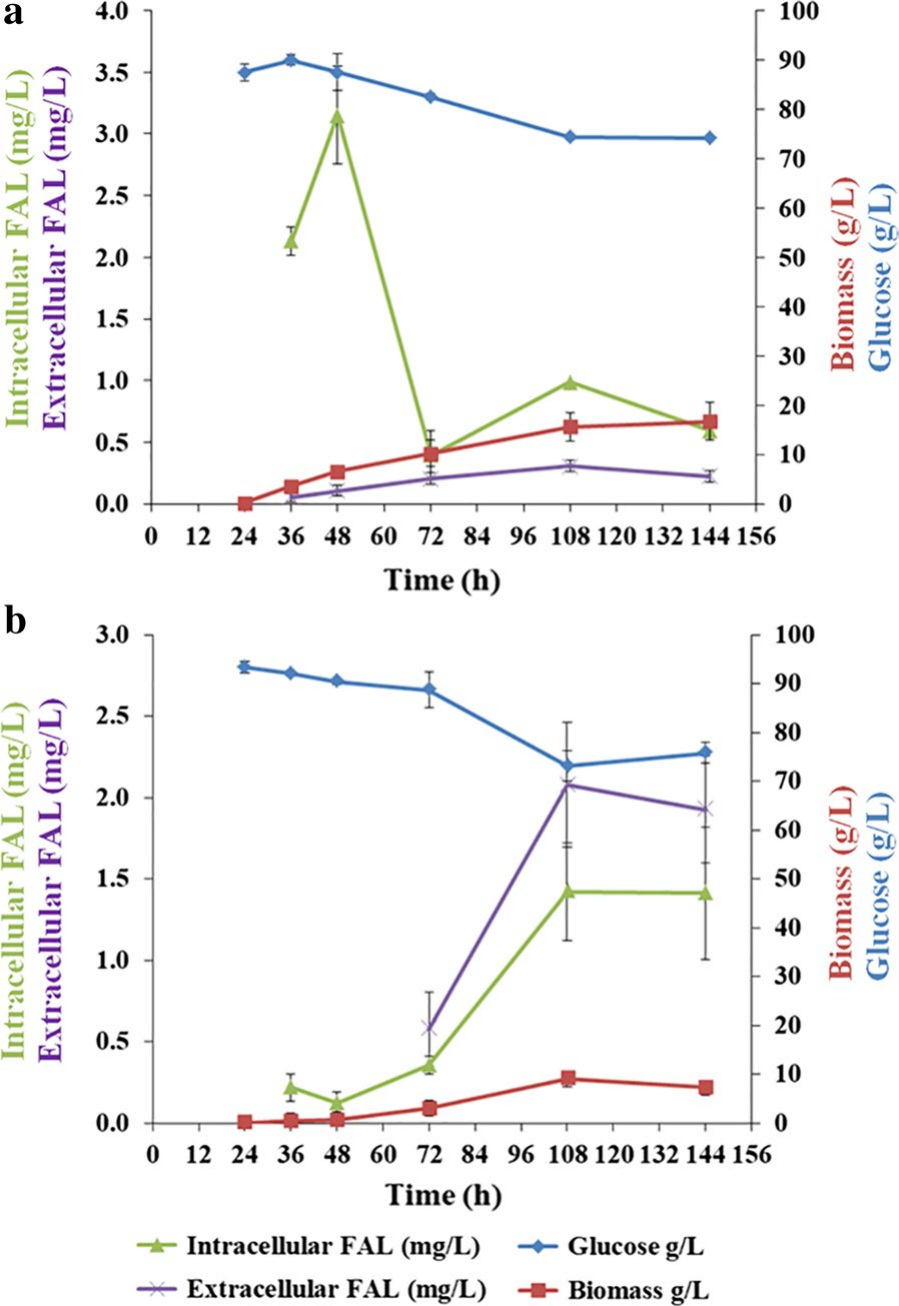
Fatty alcohol production profiles of engineered *T. reesei* strain in shake-flask fermentation. Spores of the engineered strain were inoculated and cultured on medium without (**a**) or with (**b**) dodecane addition for cultivations and detections. Intracellular and extracellular fatty alcohol (FAL, hexadecanol and octadecanol), glucose, and biomass were detected over 144 h. Results are the mean of three replicates and error bars indicate standard deviations (*n* = 3 ± SD)

To confirm *Tafar1*′s functional viability and screen strains with higher *Tafar1* expression levels, the secondary screening of cultures of sorted spores (in 96-well plate format) and fatty alcohol quantification were performed. To minimize the effect of differences in transformant growth, fluorescence/OD_600_ (specific signal, arbitrary unit) was used as an indicator of expression level, which ranged from ~ 200 to ~ 450 (Additional file 1: Table S1). Although optical density is not widely used to quantify cell growth of filamentous fungi, it can serve as an indicator for biomass estimation [36] and thus was also used in this study. Sorted strains were subsequently divided into three groups and the corresponding specific yield of fatty alcohol was tested for each of the selected strains, with ten strains per group. After 48 h cultivation on medium optimized for total fatty acid methyl ester production (Additional file 1: Fig. S3), the hexadecanol yield of selected transformants ranged from 0.8 to 2 mg/g biomass (Fig. 6a, b), which is comparable to or higher than the initial fatty alcohol-producing *Yarrowia lipolytica* strain (~ 1.7 mg/g biomass) [34]. The hexadecanol yield also showed to increase alongside elevating relative fluorescence values (Fig. 5a, b), suggesting that hexadecanol producing capability, which was tightly dependent on the FAR gene expression level [34], was highly relevant to the GFP signal intensity. The correlation between specific fluorescence and the expression level of *Tafar1* gene was also demonstrated (Fig. 5c), supporting the feasibility of screening transformants for high gene expression levels based on GFP fluorescence.

### Evaluation of the engineered *T. reesei* strain using shake-flask fermentation

The performance of the engineered *T. reesei* strain T3, with high *Tafar1* expression (Fig. 5), was preliminarily evaluated using shake-flask fermentation. Akin to most eukaryotic cell factories for fatty alcohol production [34, 37, 38], the majority of produced fatty alcohols were retained inside the cell instead of being secreted to the extracellular space (Fig. 6a). The production of both intracellular and extracellular fatty alcohols (hexadecanol and octadecanol) also showed to increase alongside biomass accumulation, achieving a maximum titer of 3.98 (intra) + 0.15 (extra) mg/l at 48 h (Fig. 6a), and then dropping to the range of 0.39–0.99 (intra) + 0.20–0.31 (extra) mg/l (Fig. 6a). This decrease in fatty alcohol production at the late-stage fermentation suggests the existence of fatty alcohol degradation pathway in *T. reesei* (potential executors include aldehyde and alcohol dehydrogenases such as Trire2_2038, Trire2_56839, and Trire2_66827), which also warrants further investigation to improve metabolic engineering of this organism for fatty alcohol production.

Overlaying dodecane was previously demonstrated as an efficient approach to recover intracellular retained fatty alcohols in yeast *S. cerevisiae* [38, 39], and was, therefore, applied here to evaluate its equivalent suitability for fatty alcohol-producing *T. reesei*. Dodecane addition inhibited biomass accumulation, resulting in a decrease in the final biomass (144 h) from 16.72 ± 3.82 g/l (Fig. 6a) to 7.24 ± 0.74 g/l (Fig. 6b). This result is dissimilar to that for *Y. lipolytica*, wherein no inhibitory effects on growth were observed and ~ 95% intracellular fatty alcohols were recovered [38]. Here, fatty alcohol distribution to the dodecane phase was 59 ± 11% for *T. reesei* cells (Fig. 6b), with the relatively lower ratio most likely being a result of intertwined hyphae limiting the accessibility of cells to dodecane. Overall, maximum fatty alcohol production was observed after 144 h cultivation, being 1.82 (intra) + 2.34 (extra) mg/l (Fig. 6b).

## Discussion

Compared to other microorganisms with regular cell shapes, the time-consuming and effort-intensive genetic engineering processes associated with *T. reesei* (Fig. 1), due to its intertwined multinucleate hyphae, thwarts strain improvement for this fungus, and its application in lignocellulose-based biorefinery. In this study, a GFP-fusion coupling flow cytometry sorting platform was established to simplify the genetic engineering procedure of *T. reesei* (Fig. 1). This enabled the rapid characterization of basic promoter elements for the manipulation of *T. reesei*, a reduction in efforts for purifying transformants functionally expressing heterologous genes and the rapid selection of transformants with high gene expression levels from a larger candidate pool.

It is clear that the versatile and high-throughput attributes of flow cytometry sorting platforms can accelerate the characterization of elements essential for genetic engineering filamentous fungi. Currently, genetic engineering efforts using filamentous fungi involve DNA segments being randomly integrated into the chromosome due to the unavailability of stable plasmids. As well, to characterize basal promoter elements, in particular the driving strengths of homologous and heterologous promoters, real-time PCR is principally used since convenient and reliable plasmid-based enzymatic assay for *E. coli* [40] and *S. cerevisiae* [41] are not suitable. Finally, the assessment of heterologous promoters can be extremely complex considering that insertion sites can have significant impacts on gene expression strength [42, 43]. Flow cytometry analysis of transformant spores offers an efficient approach to constitutive promoter strength evaluation as the results generated reflect a fluorescence value distribution for the whole-cell population (> 100,000 cells). This analysis on considerable amount of variants inserted with expression cassettes at different genomic sites would evaluate the performance of overall cell populations, and, therefore, allows for the promoter evaluation regardless of influence from various insertion sites. Furthermore, the consistency of our promoter strength results from this study (Ppki and Ptef1, Fig. 3) with that of previously reported transcriptome data also supports the feasibility of flow cytometry analysis for constitutive promoter strength evaluation.

Functional expression of heterologous genes is the prerequisite for cell factory construction, as it is essential for non-native pathway construction. Gene expression is tightly controlled in biological systems at multiple levels including transcription, translation, protein folding, and modification, which can individually or collectively hinder the successful expression of heterologous genes in various host organisms [44–46]. Although codon optimization provides a popular approach for improving gene expression levels, through relieving transcriptional inhibition and increasing translation efficiency [47, 48], the expressibility of codon optimized heterologous genes is still sometimes questioned [34]. Confirmative detection of heterologous product, therefore, always serves as the most reliable approach to evaluate heterologous genes’ expressibility. With the GFP-fusion coupling FACS platform, candidate heterologous genes were fused with a GFP-encoding gene at the 3′ termini (Fig. 1). Such construct, unlike that at the 5′ termini, allows for a robust expressibility evaluation as correct GFP translation at C-termini of the fusion protein will act as the final step of the successful expression. Fluorescence derived from GFP indicated that the protein product from the given heterologous gene has gone through the homologous control systems, suggesting the gene’s functional viability in *T. reesei* (Figs. 2, 4, 5).

For metabolic engineering *T. reesei*, and filamentous fungi in general, two additional laborious and time-consuming processes are also necessary, in comparison to similar work using model organisms such as *E. coli* or *S. cerevisiae*. This being spore separation-mediated purification and screening for strains with high expression levels. Both processes can be easily overcome with the GFP-fusion coupling FACS platform established in this work (Fig. 1), as this platform allows for (i) the analysis of transformant spores harboring target gene–*gfp* constructs (Fig. 2), and (ii) the identification and sorting/purification of transformants with successful heterologous gene expression (Figs. 4, 5). Coupled with second screening based on the fluorescence value of the pre-selected culture, this application was able to increase the coverage of the candidate transformant library, making it possible to obtain transformants with higher gene expression level (Fig. 1), whilst meanwhile greatly simplifying and shortening the build-test process in *T. reesei* (from 2 months to 2 weeks, Fig. 1). Although the time scale remains longer in comparison to using *E. coli* or *S. cerevisiae*, the high-throughput workflow which we propose in this study makes *T. reesei* a much more attractive alternative to such model organisms for bioprocessing, considering its hyper lignocellulolytic capability.

The fatty alcohol-producing *T. reesei* strain constructed in this study demonstrates comparable hexadecanol yield to the initial fatty alcohol cell factory of oleaginous *Y. lipolytica*, which remains one of the fatty alcohol producers with highest titer and yield after multi-round engineering [34]. Although the engineered *T. reesei* strain in this work cannot convert cellulose to fatty alcohols (data not shown), due mainly to the low cellulolytic capacity of strain TU-6 for insufficient nutrient supply to support cell growth, the fatty alcohol production pattern when the carbon source glucose (Fig. 6) was used, nonetheless, aided the understanding of fatty acid metabolism in *T. reesei* and, moreover, its fatty alcohol-producing capacity.

It is clear that filamentous fungi have great potential in the future as efficient cell factories for fatty acid derivatives. Furthermore, their exploitation in this capacity could be expedited by applying sophisticated tools for genetic engineering, such as the GFP-fusion coupling FACS platform we propose in this study.

As a proof-of-concept of the GFP-fusion coupling FACS platform in metabolic pathway construction, we employed single-gene engineering. Nevertheless, combinatorial utilization of additional fluorescent proteins using this platform could hold greater promise for improving multi-step metabolic pathway function in filamentous fungi.

## Conclusion

In summary, a GFP-fusion coupling FACS platform was constructed to advance the metabolic engineering of filamentous fungi, in particular the build and test process of the DBTL cycle, by bypassing the laborious verification of gene expressibility, spore separation, and transformant screening. As a proof-of-concept, the first fatty alcohol-producing filamentous fungi cell factory was constructed.

## Methods

### Strains and culture condition

*Escherichia coli* strain DH5α was used as the host strain for recombinant plasmid construction. *T. reesei* strain QM9414 (ATCC 26921) and a uridine auxotrophic *T. reesei* strain TU-6 (ATCC MYA256) were used in this study. Minimal medium (MM) [49] was used to screen and culture positive *T. reesei* transformants. It also served as the basal medium for culturing optimization of *T. reesei* for fatty acid methyl esters and fatty alcohol production. For maintenance and culturing of TU-6, 10 mM uridine was added into the medium.

### Plasmid construction

*Trpdi2*-*gfp* segment was constructed using overlap PCR following procedures previously reported [50], with Trpdi2-gfp-1F/Trpdi2-gfp-1R (*Trpdi2* part, see Trire2 gene accession number in Additional file 1: Table S2 for genes from *T. reesei*) and Trpdi2-gfp-2F/Trpdi2-gfp-2R (*gfp* part, GeneBank accession number APQ46081) listed in Table 1 as primers. *Trpdi2*-*gfp* segment was then digested with *Xba*I and *Nsi*I and inserted into digested pRLMex 30 (promoter of pyruvate kinase, no selective marker [51]) to generate p30-*Trpdi2*-*gfp* plasmid.

**Table 1:** Primers used in expression cassette construction in this study

Primer	Sequence	Comments
pyr4-cass-F	TCTAGATATCGGATCCATCCCGGCTTGCGCTTGGACCTCGC	*pyr4* expression cassette
pyr4-cass-R	ACTAGTAAGCTTCCTAGGATATGGAAGCTGATATCGTCGACAA	
TrPDI2-egfp-1F	AATCTAGAATGGTCTTGATCAAGAGCCT	*Trpdi2*-*gfp* segment
TrPDI2-egfp-1R	AACAGCTCCTCGCCCTTGCTCACCAGCTCGTCCTTCTGGTCCTCGT	
TrPDI2-egfp-2F	ACGAGGACCAGAAGGACGAGCTGGTGAGCAAGGGCGAGGAGCTGTT	
TrPDI2-egfp-2R	ATATGCATTTACTTGTACAGCTCGTCCAT	
P19-Ppdc-F	CGGTACGCGCGGATCTTCCAGAGATTCTAGATATCCGCTAGCAGGACTTCCAGGGCTACTTGGCGCG	*pdc* promoter
Ppdc-gfp-R	TGAACAGCTCCTCGCCCTTGCTCACGTCGACCTTGGGCCCCTGCAGAAGCTTCATGATTGTGCTGTAGCTGCGCTGCTTT	
Ppdc-gfp-F	AAAGCAGCGCAGCTACAGCACAATCATGAAGCTTCTGCAGGGGCCCAAGGTCGACGTGAGCAAGGGCGAGGAGCTGTTCA	*gfp*
gfp-Tcbh1-R	CACTGGCCGTAGTGAGACTGGGTAGCCATGGCTCGAGTTACTTGTACAGCTCGTCCATGCCG	
gfp-Tcbh1-F	CGGCATGGACGAGCTGTACAAGTAACTCGAGCCATGGCTACCCAGTCTCACTACGGCCAGTG	*cbh1* terminator
Tcbh1-P19-R	GTTTGCACGCCTGCCGTTCGACGATACTAGTCCTAGGTGGCCTCGCAACGGACAAGTTGGTC	
P19-Ptef1-F	CGGTACGCGCGGATCTTCCAGAGATTCTAGATATCCGCTAGCGGGACAGAATGTACAGTACTATACT	*tef1* promoter
Ptef1-gfp-R	TGAACAGCTCCTCGCCCTTGCTCACGTCGACCTTGGGCCCAAGCTTCATTTTGACGGTTTGTGTGATGTAGCGT	
Ptef1-gfp-F	ACGCTACATCACACAAACCGTCAAAATGAAGCTTGGGCCCAAGGTCGACGTGAGCAAGGGCGAGGAGCTGTTCA	*gfp*
gfp-Tegl1-R	ACGTGCACGTCTTGCACCCGCTGTACTGCAGCTCGAGGTCGACTTACTTGTACAGCTCGTCCATGCCG	
gfp-Tegl1-F	CGGCATGGACGAGCTGTACAAGTAAGTCGACCTCGAGCTGCAGTACAGCGGGTGCAAGACGTGCACGT	*egl1* terminator
Tegl1-P19-R	GTTTGCACGCCTGCCGTTCGACGATACTAGTCCTAGGTGCATTTCAAGGGCGTTGCTGAGAG	
P19-Ppki-F	CGGTACGCGCGGATCTTCCAGAGATTCTAGATATCCGCTAGCATAACGGTGAGACTAGCGGCCGGTC	*pki* promoter
Ppki-gfp-R	TGAACAGCTCCTCGCCCTTGCTCACGTCGACCTTGGGCCCCTGCAGAAGCTTCATGGTTAAGAGGGTTCTTCCGGCTTCG	
Ppki-gfp-F	CGAAGCCGGAAGAACCCTCTTAACCATGAAGCTTCTGCAGGGGCCCAAGGTCGACGTGAGCAAGGGCGAGGAGCTGTTCA	*gfp*
gfp-Tcbh2-R	TGTTTGAAGCCCGGTCACGAAAGCCCCATGGCTCGAGTTACTTGTACAGCTCGTCCATGCCG	
gfp-Tcbh2-F	CGGCATGGACGAGCTGTACAAGTAACTCGAGCCATGGGGCTTTCGTGACCGGGCTTCAAACA	*cbh2* terminator
Tcbh2-P19-R	GTTTGCACGCCTGCCGTTCGACGATACTAGTCCTAGGAAGAGGTGGAGTAATTGGAATCTAC	
Atfar1-gfp-*Pst*I-F	AAGGCCTGCAGATGGAATCCAATTGTGTTCAATTTC	*Atfar1*
Atfar1-gfp-*Sal*I-R	AACCTGTCGACTTGTTTAAGCACATGGGTGATGAGG	
Atfar6-gfp-*Pst*I-F	AAGGTCTGCAGATGTGTTTTTATGGTGAGACGTCTT	*Atfar6*
Atfar6-gfp-*Sal*I-R	AACCTGTCGACCTCAGTCTTCTTCTTAGAAAGAAAT	
Tafar-gfp-*Hin*dIII-F	GGCCTAAGCTTATGGTGTCCATCCCCGAGTACTACG	*Tafar1*
Tafar-gfp-*Sal*I-R	AACCTGTCGACGTATCGCATGGTGGAAGAGGCTCGA	
Tef1-RT-F	GCTCTGCTCGCCTACACCCT	Real-time PCR
Tef1-RT-R	TCTCCTTCTCCCAGCCCTTG	
Tafar1-RT-F	CCGACCCAACACCTACAC	
Tafar1-RT-R	GGTCCGTTAAAGTTGTCAATC	

To overcome the issue of lacking enzyme restriction sites in the previously reported plasmids (such as pRLMex 30) for gene expression in *T. reesei* and to develop basal tools for constructing strains for FACS platform analysis, a series of plasmids were constructed: p19-Ppki-*gfp*-Tcbh2, p19-Ppdc-*gfp*-Tcbh1, p19-Ptef1-*gfp*-Tegl1, p19-Ppki-*hph*-Tcbh2, and p19-*pyr4* (expression cassette of gene encoding orotidine-5′-phosphate decarboxylase). All elements were obtained through PCR with primers listed in Table 1 to introduce enzyme restriction site as the overlapping region at the end of the segments. Corresponding segments were integrated into pSIMPLE-19 *Eco*RV/BAP vector (Takara Biotechnology, Dalian, China) to form the plasmids using pEASY-Uni Seamless Cloning and Assembly Kit (Transgen Biotech, Beijing, China). The addition of restriction sites allowed insertion of target genes between promoter and *gfp* marker and easy transmission of all the elements including promoter, gene, and terminator. The biobrick design (addition of isocaudomer sties *Xba*I, *Avr*II, *Nhe*I, and *Spe*I) may also be used in the assembly of multiple expression cassettes.

To reconstruct the pathway for fatty alcohol production in *T. reesei*, the genes were cloned from plant *A. thaliana* and barn owl *T. alba* encoding fatty acyl-CoA reductases, which directly catalyze the synthesis of fatty alcohol from fatty acyl-CoA. *Atfar1* (GenBank accession number EU280149) and *Atfar6* (GenBank accession number NM_115529) segments were obtained by PCR with *A. thaliana* cDNA as template and primers, respectively, listed in Table 1, and were digested with *Pst*I and *Sal*I. Synthesized *T. alba Tafar1* (GenBank accession number JN638549) was digested with *Hin*dIII and *Sal*I. The digested *Atfar1*, *Atfar6,* and *Tafar1* were then separately inserted into digested p19-Ppdc-*gfp*-Tcbh1 to generate plasmids p19-Ppdc-*Atfar1*-*gfp*-Tcbh1, p19-Ppdc-*Atfar6*-*gfp*-Tcbh1, and p19-Ppdc-*Tafar1*-*gfp*-Tcbh1.

### Construction of *T. reesei* strains

*Trichoderma reesei* strain expressing *Trpdi2*-*gfp* was constructed through co-transformation of p30-*Trpdi2*-*gfp* and *pyr4*-pBluescript (plasmid harboring gene encoding orotidine-5′-phosphate decarboxylase) into TU-6 as described previously [49, 52]. *Trpdi2*-*gfp*-TU-6 strain and corresponding control strain *pyr4*-TU-6 were obtained traditionally after genome PCR and spore separation. *T. reesei* strains expressing *gfp* under different promoters were constructed by co-transformation of p19-*pyr4* with p19-Ppki-*gfp*-Tcbh2, p19-Ppdc-*gfp*-Tcbh1, or p19-Ptef1-*gfp*-Tegl1 plasmids into TU-6, respectively. To test the functional viability, *T. reesei* strains expressing *far*-*gfp* were constructed through co-transformation of p19-*pyr4* with p19-Ppdc-*Atfar1*-*gfp*-Tcbh1, p19-Ppdc-*Atfar6*-*gfp*-Tcbh1, or p19-Ppdc-*Tafar1*-*gfp*-Tcbh1 plasmids into TU-6, respectively. The *pdc* promoter was used for its high driving strength under high glucose concentration, which is beneficial for fatty acid derivative induction due to the high C/N ratio. Putative transformants of *T. reesei* strains expressing *gfp* or *far*-*gfp* (40 for each transformation) were transferred from selective medium (MM) with sorbitol to MM for accumulating spores (transformant pool), which were collected for subsequent analysis and sorting by flow cytometry.

### Flow cytometry analysis and cell sorting

Spores (~ 10^6^/ml) prepared by collection from MM culture and the following filtration through four-layer lens cleaning paper, or protoplast (~ 10^6^/ml) obtained by enzymatic treatment of fresh hyphae (detailed in Additional file 1: Methods) were used for analysis and cell sorting with flow cytometry using a MoFlo™ XDP cell sorter (Beckman Coulter Inc., Brea, California, USA). Analysis and cell sorting with flow cytometry were performed at a rate of 20,000 events per second. An initial scatter-gating step was conducted based on cell’s forward-scatter properties to collect data from single cells. The 488 nm laser was used to excite the GFP protein and 528/29 filter was used to detect the fluorescence signal. Flow cytometry analysis was performed by analyzing 100,000 cells of each sample. A gate was set to define positive spores expressing *gfp* or *far*-*gfp* based on the difference of their fluorescence value from reference spores (*pyr4*-TU-6). FACS was performed for the gate-defined spores expressing *gfp* and *Tafar1*-*gfp* and cell sorting of *pyr4*-TU-6 spores was carried out randomly for the whole spore population. Spores were sorted into 96-well clear-bottom black plates (Corning, New York, USA) with 200 µl liquid MM with one spore per well. *pyr4*-TU-6 spores (Con) and spores expressing *gfp* (G6) and *Tafar1*-*gfp* were sorted into one, one, and five 96-well plates, respectively. After 7 days of culturing in darkness (28 °C, 180 rpm), a secondary screen was carried out with a microplate reader (SpectraMax M2e, Molecular Devices, Sunnyvale, CA, USA) to confirm the positive transformants and to select transformants with high expression levels according to the fluorescence/OD_600_ value. Fluorescence was measured with the excitation wavelength of 488 nm and emission wavelength of 530 nm.

### Fatty alcohol extraction and quantification

Following secondary screening, cultures showing different fluorescence values (*n* = 10 for each group) were selected and inoculated onto MM plate for spore generation. 10^7^ spores were subsequently inoculated into 5 ml modified MM (100 g/l glucose and 1 g/l ammonium sulfate) in 50 ml falcon tube and cultured at 28 °C and 180 rpm for 48 h. The cultures were collected and ~ 30 mg freeze-dried hyphae were used for intracellular fatty alcohol extraction. *T. reesei* cells were broken with a glass homogenizer for 30 s at 4 °C and cell debris was resuspended with 600 μl hexane and disrupted using glass beads with Vortex-Genie 2 (Scientific Industries, New York, USA) for 30 min. After centrifugation at 14,000*g* for 5 min, supernatant was collected for quantification with GC–FID (GC-2010, Shimadzu, Kyoto, Japan). Fatty alcohol analysis with GC–FID was performed as previously described [53].

For evaluation of strain performance with shake-flask fermentation, 10^7^ spores were inoculated into 50 ml modified MM (100 g/l glucose and 1 g/l ammonium sulfate) with or without 5 ml dodecane overlay in 250 ml shake flasks and cultured at 28 °C and 180 rpm over 144 h. Biomass was then quantified on freeze-dried hyphae of 50 ml culture. Glucose concentration was determined using high-performance liquid chromatography as previously described [54]. Intracellular fatty alcohol was extracted and quantified with the procedures described as above. Extracellular fatty alcohol from cell culture without dodecane was extracted by leaching all supernatant with 2.5 ml hexane for 1 h, and the extract was analyzed with GC–FID after centrifugation at 14,000*g* for 5 min. Extracellular fatty alcohol from cell culture with dodecane addition was quantified by GC–FID analysis of the tenfold diluted dodecane extract with hexane.

### Transcript-level quantification

Spores of randomly selected strains (T1, T2, and T3) were inoculated and cultivated as described above and 48 h cell culture was subjected to RNA extraction, reverse transcription, and quantitative PCR for *Tafar1* expression level as previously described [55].

## Additional file

**Additional file 1: Fig. S1.** An overview of current processes for genetic engineering filamentous fungi, **Fig. S2.** Quantitative analysis on fluorescence of spore populations, **Fig. S3.** Biomass and fatty acid methyl ester (FAME) accumulation in *Trichoderma reesei* strain on medium with different ratios of carbon and ammonium sources, **Table S1.** Fluorescence value/OD_600_ of sorted cell cultured for 7 day and subsequently for fatty alcohol detection, **Table S2.** Trire2 accession numbers for genes of *T. reesei* used in this study.
