# Supplementary material for: A GFP-fusion coupling FACS platform for advancing the metabolic engineering of filamentous fungi
Source: Biotechnol Biofuels. 2018 Aug 24;11:232. doi: 10.1186/s13068-018-1223-8 (PMC6109270; doi:10.1186/s13068-018-1223-8)
Supplement: Supplementary file 1 — Additional file 1: Fig. S1. An overview of current processes for genetic engineering filamentous fungi, Fig. S2. Quantitative analysis on fluorescence of spore populations, Fig. S3. Biomass and fatty acid methyl ester (FAME) accumulation in Trichoderma reesei strain on medium with different ratios of carbon and ammonium sources, Table S1. Fluorescence value/OD600 of sorted cell cultured for 7 day and subsequently for fatty alcohol detection, Table S2. Trire2 accession numbers for genes of T. reesei used in this study. [file 13068_2018_1223_MOESM1_ESM.docx]

**Additional Materials**

**Journal**: Biotechnology for Biofuels

**Title***:* A GFP-fusion coupling FACS platform for advancing the metabolic engineering of filamentous fungi

**Authors**: Guokun Wang^1^, Wendi Jia^1^, Na Chen^2^, Ke Zhang^1^, Lixian Wang^1^, Pin Lv ^1^, Ronglin He^1^, Min Wang^1^, Dongyuan Zhang^1,#^

**Affiliation and address of authors**: 1, Tianjin Institute of Industrial Biotechnology, Chinese Academy of Sciences, Tianjin, 300308, People’s Republic of China; 2, Tangshan Academy of Agricultural Sciences, Tangshan, 063001, People’s Republic of China

**Correspondence to Dongyuan Zhang**: Tel.: +86-22-84868745; Fax: +86-22-84868745; E-mail: [zhang_dy@tib.cas.cn](mailto:zhang_dy@tib.cas.cn)

**
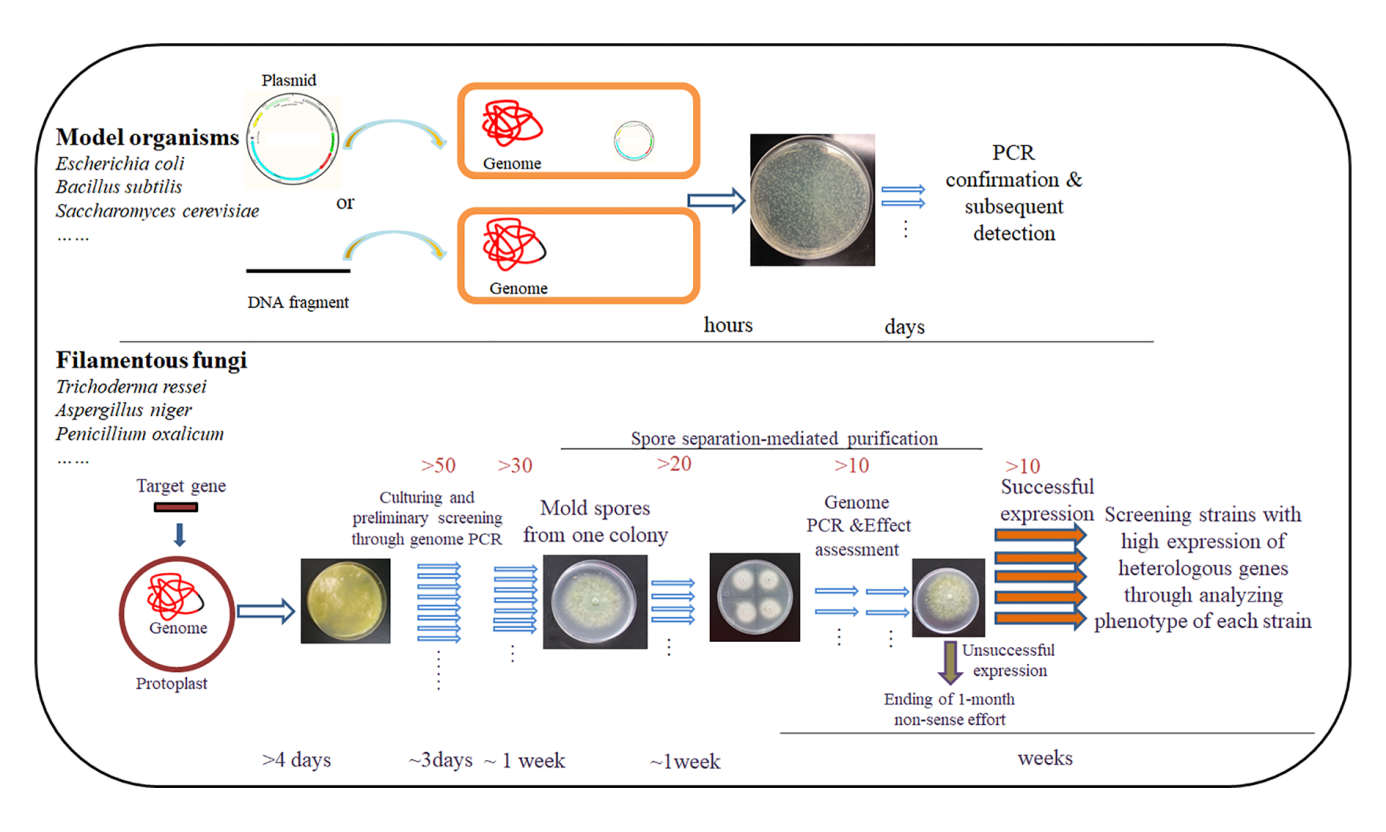
**

**Fig. S1** An overview of current processes for genetic engineering filamentous fungi

In comparison to model organisms (top) the unavailability of episomal plasmids for filamentous fungi, means that processes used for their genetic engineering can be relatively time consuming and labour intensive, with the need for genome integration of DNA fragments and spore separation-mediated purification (bottom). Time and numbers marked in the figure indicate the time and quantities of selected transformants normally required for each procedure.

The time costs for genetic engineering and evaluation of *Trichoderma reesei*, two months versus two weeks for routine procedures (shown here) and simplified processes in this study (Fig. 1A) which were indicated in this paper, was estimated based on the time for multi-round culturing and the operation for each procedures. For routine genetic procedures, eight rounds of culturing, two rounds of genomic PCR verification, real time PCR, evaluation of strains’ production and potentially repeated operations for unfortunate experimental failure will cost approximately two months for each round of genetic engineering and evaluation of the generated *T. reesei* strains. Whereas, for the simplified processes based on the platform constructed in this study, only two round culturing, flow cytometry based cell analysis and sorting and fluorescence test with microplate reader (Fig. 1A) will be needed, costing about two weeks in total for one round.


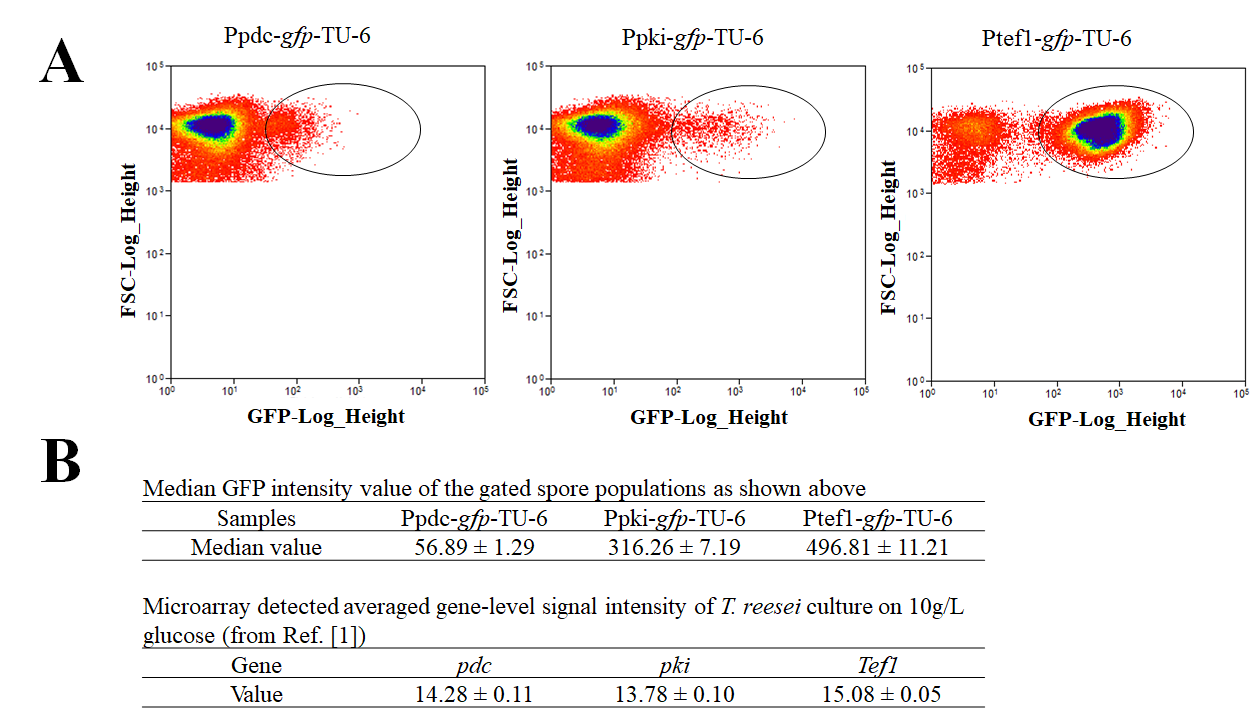


**Fig. S2** Quantitative analysis on fluorescence of spore populations

Positive GFP-expressed spore populations (separately gated for samples in panel A) under three different promoters were analyzed on the median GFP intensity value (B) for the quantitative analysis of promoter strength at spore pool level. Mean GFP intensity value and standard deviation shown here was calculated from triplicate analysis on transformant spore pools. Microarray data on gene expression was adapted from reference 1 for direct comparison of promoter strength evaluation between microarray-based and flow cytometry-based approaches.

1. Bischof R, Fourtis L, Limbeck A, Gamauf C, Seiboth B, Kubicek CP: Comparative analysis of the *Trichoderma reesei* transcriptome during growth on the cellulase inducing substrates wheat straw and lactose. Biotechnology for biofuels 2013, 6:1.


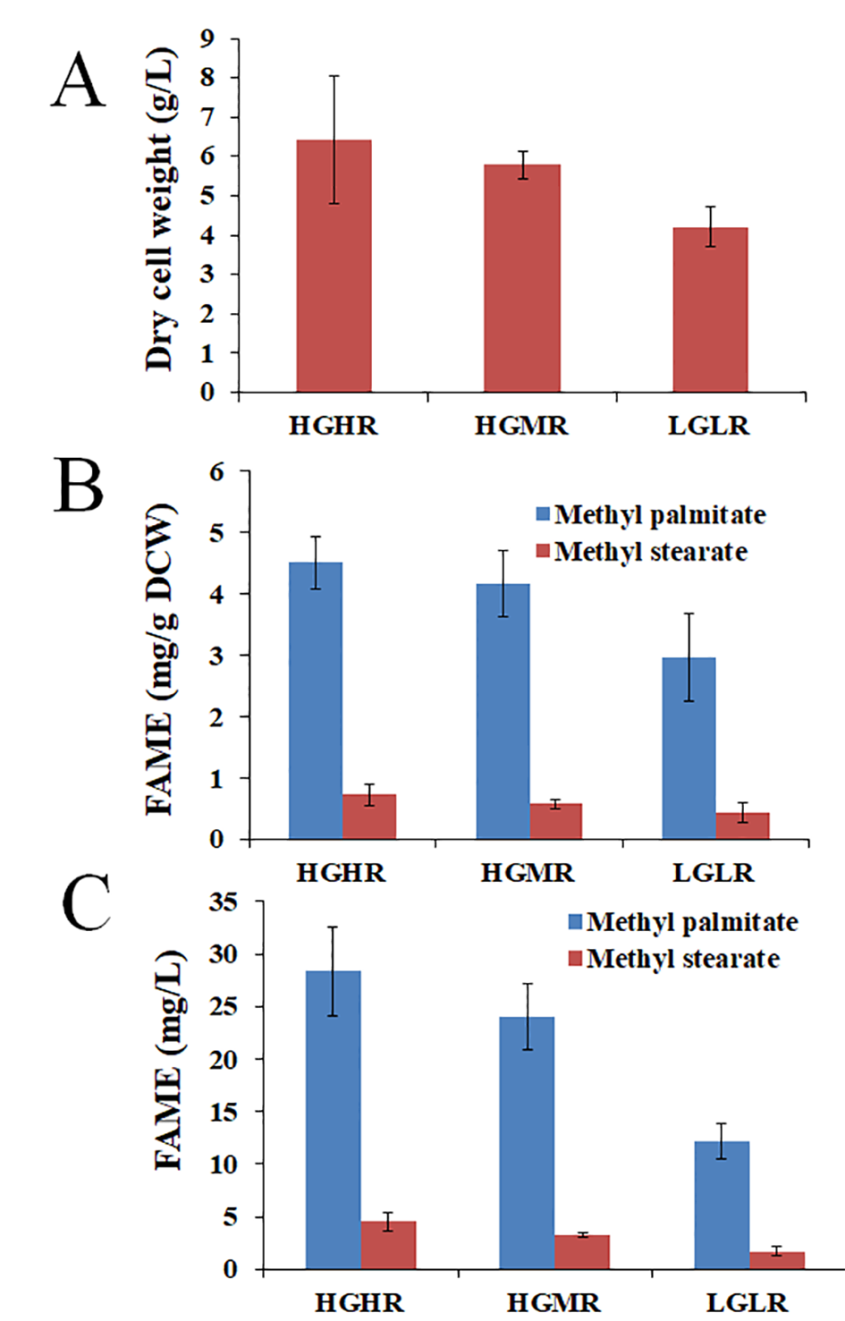


**Fig. S3** Biomass and fatty acid methyl esters (FAME) accumulation in *Trichoderma reesei* strain on medium with different ratios of carbon and ammonium sources.

*T. reesei* pyr4-TU-6 strain was cultured on minimal medium with modified contents of carbon and ammonium source for 96 h before the quantification of the dry cell weight (DCW, A) and FAME (B and C). Based on FAME production, HGHR was selected for strain cultivation for fatty alcohol production in this study.

Glucose/Ammonium sulfate ratios of 100:1, 100:6 and 10:6 were used in HGHR (high glucose concentration, high C/N ratio), HGMR (high glucose concentration, modest C/N ratio) and LGLR (low glucose concentration, low C/N ratio) respectively.

Table S1 Fluorescence value/OD600 of sorted cell cultured for 7 day and subsequently for fatty alcohol detection

| Fluorescence value/OD600 | | |
| --- | --- | --- |
| Group 200-300 | Group 300-400 | Group 400-450 |
| 222.12 | 345.40 | 435.00 |
| 295.73 | 332.53 | 410.70 |
| 242.24 | 328.68 | **448.51*** |
| 229.00 | 340.04 | 439.44 |
| **255.29*** | 309.89 | 410.96 |
| 259.34 | **345.25*** | 425.60 |
| 290.60 | 305.72 | 445.48 |
| 230.52 | 349.55 | 435.83 |
| 243.66 | 345.23 | 428.84 |
| 272.89 | 361.86 | 422.12 |

*: Fluorescence value/OD600 data for strains T1, T2 and T3 in Figure 5 respectively.

Table S2 Trire2 accession numbers for genes of *T. reesei* used in this study

| Genes | Trire2 accession number* |
| --- | --- |
| *Trpdi2* | 119890 |
| *tef1* | 46958 |
| *pdc* | 121534 |
| *pki* | 78439 |
| *cbhI* | 123989 |
| *eglI* | 122081 |
| *cbhII* | 72567 |
| *pyr4* | 74020 |

*: More information is available on <http://genome.jgi-psf.org/Trire2/Trire2.home.html>.

**Additional methods**

Protoplast preparation

Fresh spores of *Trpdi2*-*gfp*-TU-6 strain were harvested with 15 ml water (for one fully sporulated plate) and filtered through four-layer lens cleaning paper, and 50 µl spore solution was streaked on cellophane covered MEX plate (3 % Malt Extract (Sigma-Aldrich), 2 % agar (Sigma-Aldrich)). After 20 h cultivation at 28 °C, cell cultures on cellophane of 10 plates were treated with 20 ml enzyme solution (0.1 M KH_2_PO_4_, 1.2 M sorbitol, pH 5.6) with 5 mg/l lysing enzymes (from Trichoderma harzianum, Sigma-Aldrich) for 90 min at 28 °C. The resulting protoplast suspension was then filtered through four layers of lens cleaning paper. The clear protoplast filtrate was concentrated by centrifugation at 2000 rpm and 4 °C for 10 minutes and resuspension with 4 ml solution of 50 mM CaCl_2_, 1 M sorbitol and 10 mM Tris-HCl (pH 7.5) to generate the protoplast sample for flow cytometry analysis.

Fatty acid methyl esters extraction and quantification:

Cellular fatty acid amount was quantified on the fatty acid methy esters (FAME) which were converted from fatty acid. Approx. 30 mg freeze-dried hyphae broken with a glass homogenizer for 30 s at 4 °C and cell debris was used for FAME extraction and quantification following procedures previously reported in [[1](#_ENREF_1)].

Reference

1. Zhang K, Li H, Chen W, Zhao M, Cui H, Min Q, Wang H, Chen S, Li D: **Regulation of the Docosapentaenoic Acid/Docosahexaenoic Acid Ratio (DPA/DHA Ratio) in *Schizochytrium limacinum* B4D1.** *Appl Biochem Biotechnol* 2017, **182:**67-81.
